# Seawater usable for production and consumption of hydrogen peroxide as a solar fuel

**DOI:** 10.1038/ncomms11470

**Published:** 2016-05-04

**Authors:** Kentaro Mase, Masaki Yoneda, Yusuke Yamada, Shunichi Fukuzumi

**Affiliations:** 1 Department of Material and Life Science, Graduate School of Engineering, Osaka University, ALCA and SENTAN, Japan Science and Technology Agency (JST), Suita, Osaka 565-0871, Japan; 2 Department of Applied Chemistry and Bioengineering, Graduate School of Engineering, Osaka City University, Osaka 558-8585, Japan; 3 Faculty of Science and Technology, Meijo University, ALCA and SENTAN, Japan Science and Technology Agency, JST, Shiogamaguchi, Tenpaku, Nagoya, Aichi 468-8502, Japan; 4 Department of Chemistry and Nano Science, Ewha Womans University, Seoul 120-750, Korea

## Abstract

Hydrogen peroxide (H_2_O_2_) in water has been proposed as a promising solar fuel instead of gaseous hydrogen because of advantages on easy storage and high energy density, being used as a fuel of a one-compartment H_2_O_2_ fuel cell for producing electricity on demand with emitting only dioxygen (O_2_) and water. It is highly desired to utilize the most earth-abundant seawater instead of precious pure water for the practical use of H_2_O_2_ as a solar fuel. Here we have achieved efficient photocatalytic production of H_2_O_2_ from the most earth-abundant seawater instead of precious pure water and O_2_ in a two-compartment photoelectrochemical cell using WO_3_ as a photocatalyst for water oxidation and a cobalt complex supported on a glassy-carbon substrate for the selective two-electron reduction of O_2_. The concentration of H_2_O_2_ produced in seawater reached 48 mM, which was high enough to operate an H_2_O_2_ fuel cell.

## Abstract (web summary)

The generation and storage of energy from renewable sources with robust and accessible technology is of significant and growing concern. Here, the authors demonstrate the solar generation of hydrogen peroxide from seawater as a viable route towards this important goal.

## Graphical abstract

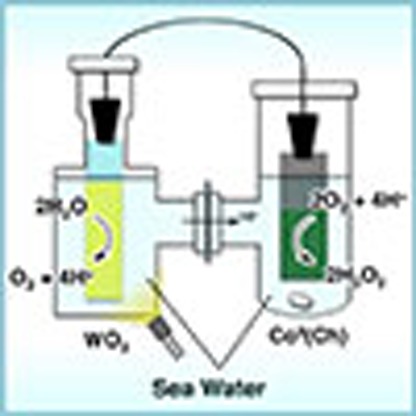

Utilization of solar energy as a primary energy source has been strongly demanded to reduce emissions of harmful and/or greenhouse gases produced by burning fossil fuels. However, large fluctuation of solar energy depending on the length of the daytime is a serious problem^1^,^2^. To utilize solar energy in the night time, solar energy should be stored in the form of chemical energy and used as a fuel to produce electricity. In this context, H_2_ has been regarded as the most promising candidate, because H_2_ can be produced by photocatalytic water splitting and used as a fuel of H_2_ fuel cells to generate electricity with a high efficiency without emission of harmful chemicals. However, the low solar energy conversion efficiency of H_2_ production and the storage problem of gaseous H_2_ have precluded the practical use of H_2_ as a solar fuel^3^. In contrast to gaseous H_2_, H_2_O_2_ can be produced as an aqueous solution from water and O_2_ in the air by the combination of the photocatalytic two-electron reduction of O_2_ and the catalytic four-electron oxidation of water^4^,^5^. H_2_O_2_ can be used as a fuel of an H_2_O_2_ fuel cell to generate electricity with emission of water and oxygen^5^,^6^,^7^,^8^,^9^,^10^. The energy density of aqueous H_2_O_2_ (60%) is 3.0 MJ l^−1^ (2.1 MJ kg^−1^), which is comparable to the value (2.8 MJ l^−1^, 3.5 MJ kg^−1^) of compressed hydrogen (35 MPa). However, the photocatalytic production of H_2_O_2_ from water and O_2_ has yet to be combined with the consumption of the produced H_2_O_2_ in an H_2_O_2_ fuel cell because of the insufficient photocatalytic activity^4^,^5^. In order to realize the production of H_2_O_2_ and its use in an H_2_O_2_ fuel cell, a breakthrough is definitely required to improve the photocatalytic efficiency for H_2_O_2_ production.

We report herein efficient photocatalytic production of H_2_O_2_, which has been made possible by using the most earth-abundant resource, that is, seawater instead of pure water for the photocatalytic oxidation with a semiconductor and the catalytic two-electron reduction of O_2_ with a cobalt chlorin complex supported on a glassy carbon substrate in a two-compartment photoelectrochemical cell under simulated solar illumination without an external bias potential. The H_2_O_2_ produced in seawater was used directly to generate electricity with the open-circuit voltage of 0.78 V and the maximum power density of 1.6 mW cm^−2^ using an H_2_O_2_ fuel cell, and the solar-to-electricity conversion efficiency of the total system is estimated to be ca 0.28%.

## Results

### Performance of photoanode and cathode

As a semiconductor photocatalyst for the water oxidation, tungsten oxide (WO_3_), which has a narrow band gap suitable for visible light (<460 nm) absorption, was employed^11^,^12^,^13^,^14^. In WO_3_, hole (h^+^) generated in the valence band (VB) is positive enough to oxidize water with the long lifetime (equation (1))^15^. The use of seawater instead of pure water has also enabled us to combine the photocatalytic production of H_2_O_2_ from seawater and O_2_ and its use in an H_2_O_2_ fuel cell. To perform selective reduction of O_2_, a cobalt chlorin


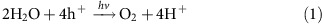


complex (Co^II^(Ch)), which has been proved to function as a catalyst for the efficient and selective two-electron reduction of O_2_ under homogeneous conditions (equation (2)), has been employed^16^. The overall photocatalytic reaction is given by equation (3), thus, H_2_O_2_ can be produced by the two-electron reduction of O_2_ by water as an electron donor.


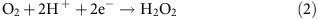



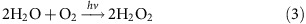


**Figure 1:**
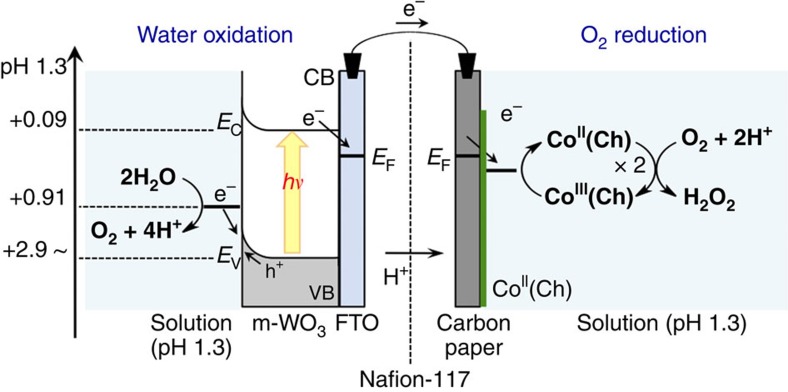
Overall scheme of photocatalytic production of H_2_O_2_. Photocatalytic production of H_2_O_2_ from water and O_2_ using m-WO_3_/FTO photoanode and Co^II^(Ch)/CP cathode in water or seawater under simulated 1 sun (AM 1.5G) illumination.

Mesoporous WO_3_ (m-WO_3_) prepared by a literature method was deposited on a fluorine-doped tin oxide (FTO) as a photoanode (m-WO_3_/FTO) and Co^II^(Ch) was adsorbed on a carbon paper (denoted as CP) as a cathode (Co^II^(Ch)/CP; see Supplementary Information for details). Photocatalytic production of H_2_O_2_ was performed by using a two-compartment photoelectrochemical cell with the m-WO_3_/FTO photoanode and the Co^II^(Ch)/CP cathode, which were immersed in Ar-saturated and in O_2_-saturated aqueous HClO_4_ solutions (pH 1.3), respectively. These two electrodes were connected to each other by a conducting wire as an external circuit. The cathode and anode cells were separated by a Nafion membrane to prevent the decomposition of H_2_O_2_ produced in the cathode cell. Overall schematic diagram is shown in Fig. 1.

To evaluate the selectivity to the two-electron reduction of O_2_ with Co^II^(Ch)/CP, the number of transferred electrons during the catalytic reaction was estimated by performing the rotating ring disc electrode (RRDE) technique with a glassy carbon disk electrode modified with a Co^II^(Ch) adsorbed on multi-walled carbon nanotubes (MWCNTs; see Supplementary Information for details). From the ratio of the observed disk current and the ring current at various rotating rates, the average number of transferred electrons was determined to be 2.7 (70% selectivity; Supplementary Fig. 1). Co^II^(Ch) has previously been reported to catalyse the production of H_2_O_2_ with nearly 100% selectivity in PhCN under homogeneous conditions^16^. The decrease in the selectivity of two-electron reduction of O_2_ may be attributed to the partial formation of μ-1,2-peroxo Co^III^(Ch) dimer, which has been reported to act as a reactive intermediate in the four-electron reduction of O_2_ (ref. 17).

### Photocatalytic production of H_2_O_2_

**Figure 2:**
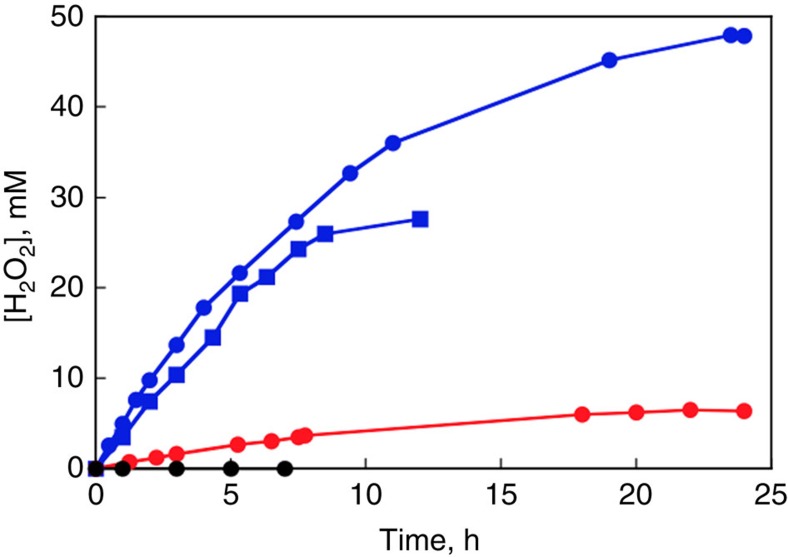
Photocatalytic production of H_2_O_2_ in the two-compartment photoelectrochemical cell. Time courses of H_2_O_2_ production with m-WO_3_/FTO photoanode and Co^II^(Ch)/CP cathode in pH 1.3 water (red circle), in pH 1.3 seawater (blue circle) and in an NaCl aqueous solution (pH 1.3; blue square) under simulated 1 sun (AM 1.5G) illumination. Time course of H_2_O_2_ production in the absence of Co^II^(Ch) on carbon paper under simulated 1 sun (AM 1.5G) illumination in pH 1.3 water is shown as black circle.

The simulated 1 sun illumination of m-WO_3_/FTO in the anode cell afforded the efficient photocatalytic production of H_2_O_2_ in the cathode cell in the two-compartment photoelectrochemical configuration without an external bias potential. The time courses of photocatalytic H_2_O_2_ production are shown in Fig. 2. Very little amount of H_2_O_2_ was obtained in the absence of Co^II^(Ch) on CP electrode, indicating that Co^II^(Ch) adsorbed on CP efficiently catalyses the two-electron reduction of O_2_ to produce H_2_O_2_ before the charge recombination of photoexcited electron in conduction band and h^+^ in VB of WO_3_. The rate of photocatalytic production of H_2_O_2_ in seawater was markedly enhanced compared with that in pure water. After the illumination for 24 h, the amount of produced H_2_O_2_ in seawater reached ca 48 mM, which is much larger than the value (2 mM) using a one-compartment system reported previously^4^. No structural change of m-WO_3_/FTO electrode after the photocatalytic reaction was confirmed by the powder X-ray diffraction measurements (Supplementary Fig. 2). The similar enhancement on photocatalytic activity was observed in an NaCl solution, in which the concentration of Cl^−^ was the same as that of Cl^−^ in seawater. The enhancement effect of Cl^−^ on the photocatalytic activity for water oxidation can be interpreted by the following Cl^−^-assisted mechanism^18^,^19^,^20^,^21^. First, the oxidation of Cl^−^ by photogenerated hole to form chlorine (Cl_2_) occurs before the oxidation of water as given by equation (4)^18^. Cl_2_ is in the disproportionation equilibrium with hypochlorous acid (HClO), as given by equation (5), where the population of Cl_2_ and HClO varies depending on the pH the solution and Cl_2_ is a major component under an acidic solution below pH 3 (refs 18, 19). Second, HClO is decomposed to O_2_ and Cl^−^ under solar irradiation, as given by equation (6)^20^. Thus, the overall reaction of the water oxidation assisted by Cl^−^ is given by equation (1). Indeed, the ultraviolet–visible absorption spectrum


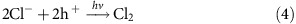



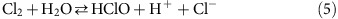



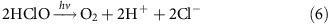


of the anode cell solution after the photocatalytic reaction for 24 h exhibited the absorption band around at 231 nm, which is identical to the spectrum of a standard HClO/Cl_2_ solution below pH 3 (Supplementary Fig. 3)^19^. The amount of O_2_ evolved in seawater in anode cell after 1 h (12.7 μmol) was more than three times larger than that in water (3.7 μmol) as shown in Supplementary Fig. 4. Thus, the enhancement of photocatalytic production of H_2_O_2_ in seawater (Fig. 2) results from the photocatalytic oxidation of Cl^−^ in seawater.

**Figure 3:**
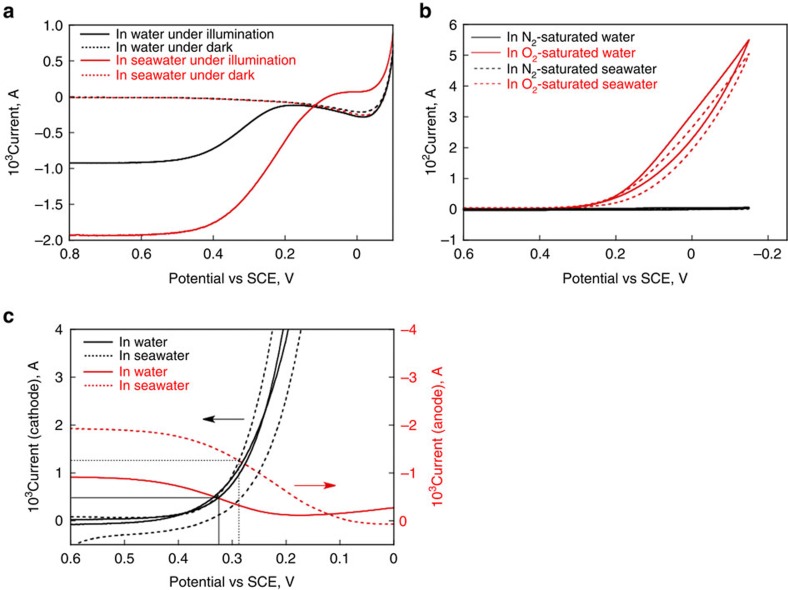
Photoelectrochemical performance of m-WO_3_/FTO and electrochemical performance of Co^II^(Ch)/CP. (**a**) *I*–*V* curves of m-WO_3_/FTO photoanode in pH 1.3 water (black solid) and in pH 1.3 seawater (red solid) under simulated 1 sun (AM 1.5G) illumination. *I*–*V* curves under dark are shown as dashed lines with the same colour definition. Sweep rate: 10 mV s^−1^. (**b**) Cyclic voltammograms of Co^II^(Ch)/CP in a N_2_-saturated pH 1.3 water (black solid) and an O_2_-saturated pH 1.3 water (red solid). The dashed lines show the cyclic voltammograms of Co^II^(Ch)/CP recorded in pH 1.3 seawater. Sweep rate: 20 mV s^−1^. (**c**) Cyclic voltammograms of Co^II^(Ch)/CP in O_2_-saturated pH 1.3 solutions (black) and *I*–*V* curves of m-WO_3_/FTO photoanode in pH 1.3 solutions (red) under simulated 1 sun (AM 1.5G) illumination.

The effects of Cl^−^ on the catalytic performance of m-WO_3_/FTO and Co^II^(Ch)/CP were also investigated by using a photoelectrochemical cell with three electrode configuration. The current–potential (*I*–*V*) curves of m-WO_3_/FTO under simulated 1 sun illumination and dark are shown in Fig. 3a. The onset of photocurrent for the oxidation of water was observed at 0.2 V (versus saturated calomel electrode (SCE)) in water (pH 1.3), which corresponds to the 110 mV of overpotential with respect to the value of flat band potential of WO_3_ (0.09 V versus SCE at pH 1.3)^22^. This overpotential is required as a driving force for the migration of the photogenerated electron from conduction band of WO_3_ to FTO electrode. When the photocatalysis measurements of m-WO_3_/FTO were performed under the same conditions in seawater instead of pure water, about four times larger photocurrent at 0.3 V (versus SCE) than that in water together with the negative shift of onset potential from 0.2 V (versus SCE) to 0.1 V (versus SCE) was observed (Fig. 3a). In addition, the stability of a photocurrent obtained by applying 0.3 V (versus SCE) was also improved as shown in Supplementary Fig. 5a. These improvements in the stability as well as the photocurrent in seawater can be explained by the efficient quenching of the photogenerated hole in VB by the oxidation of Cl^−^, as described above. These are consistent with the enhancement of photocatalytic production of H_2_O_2_ in seawater (Fig. 2). The Faradaic efficiencies in the photoelectrochemical water oxidation in water and in seawater were determined to be 77% and 93%, respectively, from the simultaneous measurements of O_2_ evolution (Supplementary Fig. 5b). The effect of Cl^−^ on the electrochemical property of Co^II^(Ch)/CP was also studied by measuring cyclic voltammograms using the three-electrode electrochemical cell. The addition of tetra-*n*-butylammonium chloride (0.1 M) to a N_2_-saturated PhCN solution containing Co^II^(Ch) (1 mM) and tetra-*n*-butylammonium hexafluorophosphate (TBAPF_6_; 0.1 M) resulted in the large negative shift of the redox potential for [Co^III^(Ch)]^+^/Co^II^(Ch) from 0.37 to 0.01 V (versus SCE; Supplementary Fig. 6a,b). In addition, no catalytic current was obtained for the O_2_ reduction in the presence of Cl^−^ in an O_2_-saturated PhCN solution containing HClO_4_ (10 mM) as a proton source (Supplementary Fig. 6c), suggesting that Cl^−^ inhibits electron transfer from Co^II^(Ch) to O_2_ because of the strong axial coordination of Cl^−^ to the reaction centre of Co^II^(Ch) to form 5- or 6-coordinated inactive species. In contrast, the catalytic current for the O_2_ reduction with Co^II^(Ch)/CP measured in seawater (pH 1.3) appeared at ca 0.34 V (versus SCE), which is virtually the same onset potential and catalytic current measured in water (pH 1.3), as shown in Fig. 3b. This result indicates that the coordination of Cl^−^ to Co^II^(Ch) adsorbed on the electrode surface in an aqueous solution is negligibly weak as compared with that in PhCN^23^. Hence, the enhancement of photocatalytic production of H_2_O_2_ is mainly derived from the acceleration of water oxidation at the photoanode. The predicted operating current of the two-compartment photoelectrochemical cell was defined from the intersection of cyclic voltammograms of Co^II^(Ch)/CP and *I–V* curves of the m-WO_3_/FTO photoanode, giving a value of 0.5 mA at 0.32 V (versus SCE) in water and 1.3 mA at 0.29 V (versus SCE) in seawater, respectively (Fig. 3c).

The effect of illumination intensity on the photocatalytic production of H_2_O_2_ was examined as shown in Supplementary Fig. 7a, where the produced amount of H_2_O_2_ increased in proportion to the intensity of the illumination. The solar energy conversion efficiency for the photocatalytic production of H_2_O_2_ in seawater was determined to be 0.55% under simulated 1 sun illumination. The best solar energy conversion efficiency was determined to be 0.94% when illumination intensity was reduced to 0.1 sun (Supplementary Fig. 7b). This efficiency exceeds that of swichgrass (0.2%), which has been considered as a promising crop for biomass fuel^24^, and also the value in the one-compartment cell (0.25%)^4^. A much higher solar-to-hydrogen efficiency of 12.3% has recently been achieved by perovskite photovoltaics-based electrolysis^2^. However, the storage of hydrogen has still been a quite difficult issue, because hydrogen is a gas having a low volumetric energy density. In this contrast, in our system, the produced hydrogen peroxide in seawater can be used directly as a fuel in an H_2_O_2_ fuel cell.

### H_2_O_2_ fuel cell

**Figure 4:**
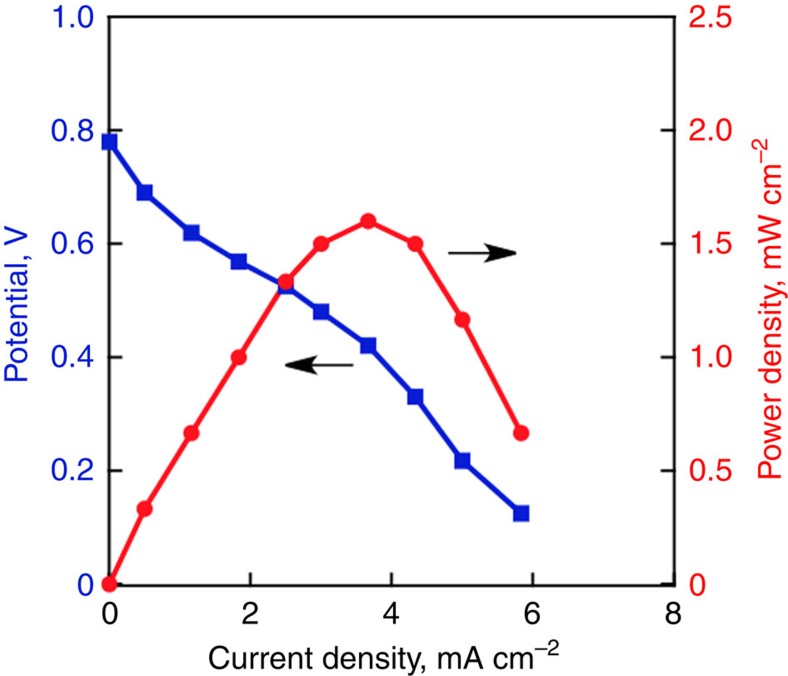
Generation of electrical energy in the one-compartment H_2_O_2_ fuel cell. *I*–*V* (blue) and *I*–*P* (red) curves of the one-compartment H_2_O_2_ fuel cell with a Ni mesh anode and Fe^II^_3_[Co^III^(CN)_6_]_2_/carbon cloth cathode in the reaction solution containing H_2_O_2_ (47.9 mM) produced by photocatalytic reaction in seawater as shown in Fig. 2 (blue circle).

Finally, the chemical energy of H_2_O_2_ produced by the photocatalytic reaction was converted to electrical energy through a H_2_O_2_ fuel cell composed of a polynuclear cyanide complexes, Fe^II^_3_[Co^III^(CN)_6_]_2_, modified carbon cloth cathode and a nickel mesh anode in a one-compartment cell^10^. The reaction solution (seawater, pH 1.3) containing ca 48 mM of H_2_O_2_ in cathode was transferred to the H_2_O_2_ fuel cell. The obtained potential and power density depending on the current density are shown in Fig. 4. The cell has the open-circuit potential and the maximum power density of 0.78 V and 1.6 mW cm^−2^, respectively. These values agree with those obtained from the H_2_O_2_ fuel cell using an aqueous solution (pH 1.0) containing authentic H_2_O_2_ (50 mM) and NaCl (1.0 M) as a supporting electrolyte (Supplementary Fig. 8). In addition, the energy conversion efficiency of the H_2_O_2_ fuel cell was determined to be ca 50% by the measurement of output energy as electrical energy versus consumed chemical energy H_2_O_2_ gas, which is comparable to the efficiency of an H_2_ fuel cell (Supplementary Figs 9 and 10). Thus, the solar-to-electricity conversion efficiency of the total system is estimated to be ca 0.28% (0.55 × 50%), which is still much lower in contrast to the conventional solar-to-electricity device such as photovoltaic cells. However, there are still many things to do in the one-compartment H_2_O_2_ fuel cells including better anode materials to improve the performance, because the one compartment cell without membrane and use of an aqueous solution of H_2_O_2_ have significant advantages as compared with H_2_ fuel cells. The production of chemical energy utilizing solar energy and its conversion to electrical energy based on H_2_O_2_ in seawater can provide practical solution to the construction of an ideal energy-sustainable society using seawater, which is the most earth-abundant resource.

## Methods

### Materials

Chemicals were purchased from commercial sources and used without further purification, unless otherwise noted. Benzonitrile (PhCN) used for spectroscopic and electrochemical measurements was distilled over phosphorus pentoxide before use^25^. Potassium hexacyanoferrate(III) (K_3_[Fe(CN)_6_]) and acetylacetone (≥99%) were purchased from Wako Pure Chemical Industries Ltd., Tungsten hexachloride (WCl_6_, ≥95%) was purchased from Nacalai Tesque. Scandium(III) nitrate tetrahydrate was purchased from Mitsuwa Chemicals. Potassium hexacyanocobaltate (K_3_[Co^III^(CN)_6_], ≥99.9%) was supplied by Stream Chemicals. Red sea salt was supplied by Red Sea. Oxo[5,10,15,20-tetra(4-pyridyl)porphinato]titanium(IV) ([TiO(tpyp)]) was purchased from Tokyo Chemical Industry Co., Ltd. (TCI). Pluronic P-123, Triton X-100, Nafion perfluorinated ion exchange resin solution, Nafion perfluorinated membrane (Nafion 117) were received from Aldrich Chemical Co. CP electrode (EC-TP1-060T produced by Toray Industry Inc.) was obtained from Toyo Co. Glass slides coated with FTO (transmittance, 83.6%) were supplied by Aldrich Chemicals Co. and cut by Asahi Glass Co., Ltd. Tetra-*n*-butylammonium hexafluorophosphate (TBAPF_6_) purchased from Wako Pure Chemical Industries, Ltd. was twice recrystallized from ethanol and dried *in vacuo* before use. Purified water was provided by a Millipore Milli-Q water purification system (Millipore, Direct-Q 3 UV) with an electronic conductance of 18.2 MΩ cm. Cobalt chlorin complex [Co^II^(Ch)], mesoporous WO_3_ (m-WO_3_) and polynuclear cyanide complex (Fe^II^_3_[Co^III^(CN)_6_]_2_) were synthesized according to the literature procedure (*vide infra*).

### Preparation of Co^II^(Ch)/CP electrode

Co^II^(Ch)/CP electrode was prepared by an MeCN solution (1 ml) of Co^II^(Ch) (0.3 mM), MWCNT (0.63 mg) and 5% Nafion (12 μl). For each experiment, the mixture was sonicated for 20 min and then a 50 μl of the mixture was applied on the both side of surface of a CP with a 3.0 cm^2^ area by drop-casting and allowed to evaporate to afford a film containing a MWCNT loading of 50 μg cm^−2^ and a catalyst loading of 30 nmol.

### Preparation of m-WO_3_/FTO electrode

First, FTO glasses were cleaned before use by immersing into an MeOH/HCl (1/1 (v/v)) solution for 30 min and washed by purified water. The resulting FTO glasses were hydroxylated in H_2_SO_4_ for 2 h and then boiled in purified water for 30 min with subsequent drying under N_2_ (ref. 26). m-WO_3_/FTO electrode was prepared by a solution consisting of 1 ml of water containing 50 mg of m-WO_3_ and acetyl acetone (30 μl) and 1 drop of Triton X-100. The mixture was sonicated for 5 min and then a 50 μl drop was applied on the surface of FTO electrode with a 2.5 cm^2^ area and allowed to evaporate to afford a thin film. The resulting electrode was annealed to form crystalline m-WO_3_ at 400 °C with ramping rate of 2 °C min^−1^ (held at 400 °C for 2 h) under air to remove surfactant species. The combustion of residual surfactant was confirmed by thermal gravimetric-differential thermal analysis (TG-DTA) of m-WO_3_ dispersion prepared above (Supplementary Fig. 11). The exothermic current peak along with weight loss observed at around 180 °C was disappeared after the annealing at 400 °C. The morphology of obtained m-WO_3_/FTO was observed by scanning electron microscope (SEM), as shown in Supplementary Fig. 12. The mesoporous structure of m-WO_3_ was confirmed by N_2_ adsorption–desorption isotherm measurements (Supplementary Fig. 13). The measurements performed at 77 K revealed a type IV isotherm^27^, clearly indicating the presence of mesopores. The Brunauer–Emmett–Teller surface area of m-WO_3_ was as high as 21 m^2^ g^−1^ (Supplementary Fig. 13a). The size of the mesopores was determined to be 8 nm by Barrett–Joyner–Halenda plot (Supplementary Fig. 13b).

### Preparation of seawater

The seawater was prepared by dissolving 33.4 g of red sea salt in 1 l of water to form a solution containing ca 550 mM of NaCl.

### Characterization of m-WO_3_/FTO

TG/DTA data were performed on an SII TG/DTA 7,200 instrument. A sample (ca10 mg) was loaded into an Al pan and heated from 25 °C to 600 °C with a ramping rate of 2 °C min^−1^ under N_2_. A certain amount of *γ*-Al_2_O_3_ was used as a reference for DTA measurements. Nitrogen-adsorption/desorption measurements were performed at 77 K on a Belsorp-mini (BEL Japan, Inc.) within a relative pressure range from 0.01 to 101.3 kPa. A sample mass of ca 100 mg used for adsorption analysis was pretreated at 150 °C for 2 h under vacuum conditions and kept in N_2_ atmosphere until N_2_-adsorption measurements. The resulting sample was exposed to a mixed gas of He and N_2_ with a programmed ratio and adsorbed amount of N_2_ was calculated from the change of pressure in a cell after reaching equilibrium (at least 5 min). Powder X-ray diffraction patterns were recorded on a Rigaku MiniFlex 600. Incident X-ray radiation was produced by a Cu X-ray tube, operating at 40 kV and 15 mA with Cu K*α* radiation (*λ*=1.54 Å). The scan rate was 1° min^−1^ from 2*θ*=10–70°. SEM images of particles were observed by a FE-SEM (JSM-6320F or JSM-6701F) operating at 10 kV.

### Electrochemical measurements

Cyclic voltammetry measurements were performed on an ALS 630B electrochemical analyser. Effects of Cl^−^ on Co^II^(Ch) was investigated in a N_2_- or O_2_-saturated PhCN solution containing 0.10 M TBAPF_6_ as a supporting electrolyte at 298 K using a conventional three-electrode cell with a glassy carbon (GC) working electrode (surface area of 0.3 mm^2^) and a platinum wire (Pt) as the counter electrode. The GC working electrode was routinely polished with polishing alumina suspension and rinsed with acetone before use. The potentials were measured with respect to the Ag/AgNO_3_ (1.0 × 10^−2^ M) reference electrode. All potentials (versus Ag/AgNO_3_) were converted to values versus SCE by adding 0.29 V (ref. 28). Redox potentials were determined using the relation *E*_1/2_=(*E*_pa_+*E*_pc_)/2.

Electrochemical performance of Co^II^(Ch) deposited on CP electrode for the catalytic O_2_ reduction was evaluated in a N_2_- or O_2_-saturated aqueous HClO_4_ (pH 1.3) solution containing NaClO_4_ (0.1 M) as a supporting electrolyte and in N_2_- or O_2_-saturated seawater containing HClO_4_ (pH 1.3) and NaClO_4_ (0.1 M) at 298 K using a conventional three-electrode cell consisting of Co^II^(Ch)/CP as a working electrode and a platinum coil as the counter electrode. All the photoelectrochemical and electrochemical measurements in aqueous solutions were conducted using a reference SCE and all results in this work are presented against the SCE. The conversion of potentials versus SCE to versus normal hydrogen electrode (NHE) was performed according to the following equation (7).


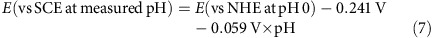


The superior performance of Co^II^(Ch)/CP in an aqueous solution was confirmed by the comparison with cobalt octaethylporphyrin (Co^II^(OEP)), which is commonly used as an electrocatalyst for the O_2_ reduction, modified CP (Co^II^(OEP)/CP), as shown in Supplementary Fig. 14.

The RRDE measurements were carried out using a BAS RRDE-3A rotator linked to an ALS 730D electrochemical analyser. A three-electrode cell (100 ml) was employed with the RRDE consisting of a platinum ring (Pt) electrode and a GC disk electrode, platinum coil (Pt) as a counter electrode and SCE as a reference electrode. The voltammograms were measured in an O_2_-saturated aqueous HClO_4_ solution (pH 1.3) containing NaClO_4_ (0.1 M) at 5 mV s^−1^ with various rotating rates (100, 300, 600, 900, 1,200, 1,500, 2,000, 2,500, 3,000, 3,500, 4,000 and 4,500 r.p.m.). A RRDE for the investigation of transferred electrons during catalytic O_2_ reduction with Co^II^(Ch)/CP was performed by the modification of GC disk electrode with a thin film of Co^II^(Ch). The thin film was prepared by a solution consisting of MeCN (1 ml) containing Co^II^(Ch) (0.3 mM), MWCNT (1.26 mg) and 5% Nafion (12 μl). For each experiment, the mixture was sonicated for 20 min and then a 10-μl drop was applied on the surface of a polished GC disk electrode and allowed to evaporate to afford a thin film containing a MWCNT loading of 100 μg cm^−2^ and a catalyst loading of 3 nmol.

The number of transferred electrons (*n*) is determined by following equation *n*=4*I*_D_/(*I*_D_+*I*_R_/*N*), where *I*_D_ is the faradic current at the disk electrode, *I*_R_ is the faradic current at the ring electrode, and *N* is the collection efficiency of the RRDE. The *N* value is measured using an aqueous solution of K_3_[Fe^III^(CN)_6_] (2 mM) as a standard one-electron redox couple ([Fe^III^(CN)_6_]^3−^/[Fe^II^(CN)_6_]^4−^) in the presence of KNO_3_ (0.5 M) and is determined to be *N*=0.37 when the GC disk electrode of RRDE is loaded with the same amount of MWCNT (100 μg cm^−2^) as used above (Supplementary Fig. 15).

### Photoelectrochemical measurements

Photoelectrochemical measurements were performed in a home-made quartz cell (light path length=1 cm) composed of the as prepared m-WO_3_/FTO electrode, a platinum coil counter electrode, and a SCE reference electrode in an Ar-saturated aqueous solution (8 ml) containing HClO_4_ (pH 1.3) and 0.1 M of NaClO_4_ at 298 K (Supplementary Fig. 16a). The photoanode was illuminated from the back side of FTO electrode (FTO/electrolyte interface) with a solar simulator (HAL-320, Asahi Spectra Co., Ltd.), where the light intensity was adjusted at 100 mW cm^−2^ (AM1.5G) at the sample position by a 1SUN checker (CS-20, Asahi Spectra Co., Ltd.). The Faradaic efficiency for O_2_ evolution was determined by following equation (8), where *F* denotes Faradaic constant (9.65 × 10^4^ C mol^−1^).

The Faradaic efficiency for O_2_ evolution (%)


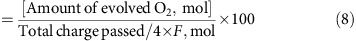


### Photocatalytic production of H_2_O_2_

Photocatalytic production of H_2_O_2_ was performed in a quartz anode cell (light path length=1 cm) connected with a pyrex cathode cell through a Nafion membrane (Supplementary Fig. 16b). The anode cell consists of the as prepared m-WO_3_/FTO photoanode for the water oxidation in an Ar-saturated aqueous solution (8 ml) containing HClO_4_ (pH 1.3) and 0.1 M of NaClO_4_. The cathode cell is composed of the as prepared Co^II^(Ch)/CP cathode for the O_2_ reduction in an O_2_-saturated aqueous solution (10 ml) containing HClO_4_ (pH 1.3) and 0.1 M of NaClO_4_ at 298 K. These two electrodes were connected each other with alligator clips and copper wire as an external circuit. The photoanode was illuminated from the back side of the FTO electrode with the solar simulator (HAL-320, Asahi Spectra Co., Ltd.), where the light intensity was adjusted at 100 mW cm^−2^ (AM1.5G) at the sample position by the 1SUN checker (CS-20, Asahi Spectra Co., Ltd.). The anode and cathode solution was saturated by continuous bubbling with argon and oxygen gas for 30 min, respectively, before the photocatalytic reaction. The O_2_ bubbling was continued during the photocatalytic reaction. The cathode cell was kept in dark to prevent the decomposition of produced H_2_O_2_ by ultraviolet-light irradiation during photocatalytic reaction. The amount of produced H_2_O_2_ was determined by spectroscopic titration with an acidic solution of [TiO(tpypH_4_)]^4+^ complex (Ti-TPyP reagent)^29^. The Ti-TPyP reagent was prepared by dissolving 3.40 mg of the [TiO(tpyp)] complex in 100 ml of hydrochloric acid (50 mM). A small portion of the reaction solution was sampled and diluted with water depending on the concentration of produced H_2_O_2_. To 0.25 ml of 4.8 M HClO_4_ and 0.25 ml of the Ti-TPyP reagent, a diluted sample was added. The mixed solution was then allowed to stand for 5 min at room temperature. This sample solution was diluted to 2.5 ml with water and used for the spectroscopic measurement. The absorbance at *λ*=434 nm was measured by using a Hewlett Packard 8453 diode array spectrophotometer. A blank solution was prepared in a similar manner by adding distilled water instead of the sample solution to Ti-TPyP reagent in the same volume with its absorbance designated as *A*_B_. The difference in absorbance was determined as follows: Δ*A*_434_=*A*_B_−*A*_S_. Based on Δ*A*_434_ and the volume of the solution, the amount of H_2_O_2_ was determined (Supplementary Fig. 17).

### Detection of O_2_

The concentration of O_2_ in the anolyte was monitored during both photocatalytic production of H_2_O_2_ and photoelecrochemical measurements (*vide supra*) by using a fluorescence-based oxygen sensor (FOXY Fiber Optic Oxygen Sensor, Ocean Optics). The O_2_-sensing needle probe was installed in a gas-tight quartz anode cell filled with 8 ml of a solution, which left 7 ml of a headspace, through a rubber septum on the end of the cell. The solution and headspace were purged with argon gas for 30 min before measurements. Two-point calibration of the O_2_ sensor was performed against solutions (air, 20.9% O_2_, and Ar, 0% O_2_) used in each measurement. The amount of O_2_ leaked in the anode cell during measurements was determined under dark and subtracted from the data obtained under illumination. The amount of dissolved O_2_ in solutions was recorded as mole %. The total amount O_2_ evolved in the anode cell was determined using Henry's Law and converted, using the ideal gas law, into μmol.

### Solar-to-H_2_O_2_ energy conversion efficiency

Measurement of solar energy conversion efficiency of the photocatalytic production of H_2_O_2_ was carried out in a quartz anode cell (light path length=1 cm) connected with a pyrex cathode cell through a Nafion membrane as used in photocatalytic production of H_2_O_2_ as described above. The photoanodes were illuminated from back side of the FTO electrode with the solar simulator (HAL-320, Asahi Spectra Co., Ltd.), where the light intensity was adjusted at 10–100 mW cm^−2^ (AM1.5G) at the sample position by the 1SUN checker (CS-20, Asahi Spectra Co., Ltd.). The amount of produced H_2_O_2_ was determined by the titration with the Ti-TPyP reagent (*vide supra*). The solar energy conversion efficiency was determined by following equations (3 and 9), where output energy as H_2_O_2_ was calculated by

Solar Energy Conversion Efficiency (%)


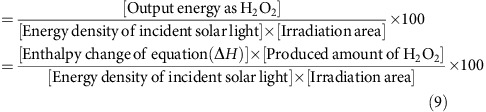


the multiplication of enthalpy change (Δ*H*=98.3 kJ mol^−1^) and the produced amount of H_2_O_2_ (the concentration of H_2_O_2_ × volume of cathode solution (10 ml)). Energy density of incident solar light was adjusted at 10–100 mW cm^−2^ (0.1–1 SUN, Air Mass 1.5 (AM1.5)) at the sample position for whole irradiation area (2.5 cm^2^) by the 1 SUN checker (CS-20, Asahi Spectra Co., Ltd.) at room temperature.

### Spectroscopic measurements

Ultraviolet–visible spectroscopy was carried out on a Hewlett Packard 8453 diode array spectrophotometer at room temperature using quartz cell (light path length=1.0 cm).

### H_2_O_2_ fuel cell

Fe^II^_3_[Co^III^(CN)_6_]_2_ was mounted onto a carbon cloth by drop-casting or by spraying a dispersion of Fe^II^_3_[Co^III^(CN)_6_]_2_ in isopropanol with an airbrush (TAMIYA Spray-work HG). An aqueous solution of Nafion (0.2 wt.%) was used to protect the film of Fe^II^_3_[Co^III^(CN)_6_]_2_ on a carbon cloth. A Ni mesh (150 mesh) and Fe^II^_3_[Co^III^(CN)_6_]_2_ that was mounted onto a carbon cloth were immersed in the solution of H_2_O_2_. The performance tests were conducted in a one-compartment cell with the reaction solution containing H_2_O_2_ produced by the photocatalytic reaction transferred from the cathode cell of the two-compartment cell system. The current and power values normalized by the geometric surface area of an electrode were recorded on an ALS 630B electrochemical analyser and KFM 2005 FC impedance meter at 25 °C. The performance tests in solutions containing various concentrations of standard H_2_O_2_, HClO_4_ (pH 1) and NaCl (1.0 M) were performed for the control experiment (Supplementary Fig. 8).

### Energy conversion efficiency of H_2_O_2_ fuel cell

Fe^II^_3_[Co^III^(CN)_6_]_2_/carbon cloth and Ni mesh electrodes were prepared as noted above. Each electrode was connected with Pt wire and protected by PP (polypropylene) sheet to avoid electrical short circuit. The performance tests were conducted in a well-sealed one-compartment cell with a rubber septum (Supplementary Fig. 9). The reaction solution containing H_2_O_2_ (0.3 M), NaCl (1.0 M) and Sc(NO_3_)_3_·4H_2_O (0.1 M)^10^ and the headspace (6.5 ml) of the one-compartment cell were purged separately with argon gas for 30 min before measurements. After the argon-saturated reaction solution was transferred to the one-compartment cell using gas-tight syringe, cell voltage, applying constant current of 3.3 mA, was recorded on a KFM 2005 FC impedance meter at 25 °C. The amount of evolved O_2_ gas in the headspace of one-compartment cell was quantified by a Shimadzu GC-17A gas chromatograph (Ar carrier, a capillary column with molecular sieves (Agilent Technologies, 19095PMS0, 30 m × 0.53 mm) at 313 K) equipped with a thermal conductivity detector. The energy conversion efficiency of H_2_O_2_ fuel cell was determined by following equations (3) and (10), where consumed chemical energy as H_2_O_2_ was calculated by the multiplication of enthalpy change (Δ*H*=−98.3 kJ mol^−1^) and twice of the produced amount of O_2_ (Supplementary Fig. 10).

Energy Conversion Efficiency of H_2_O_2_ Fuel Cell (%)


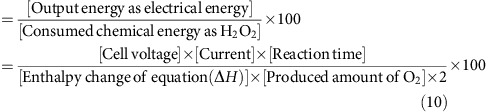


## Additional information

**How to cite this article:** Mase, K. *et al*. Seawater usable for production and consumption of hydrogen peroxide as a solar fuel. *Nat. Commun.* 7:11470 doi: 10.1038/ncomms11470 (2016).

## Supplementary Material

Supplementary Information Supplementary Figures 1-19, Supplementary Methods and Supplementary References
