# Supplementary material for: Seawater usable for production and consumption of hydrogen peroxide as a solar fuel
Source: Nat Commun. 2016 May 4;7:11470. doi: 10.1038/ncomms11470 (PMC4857479; doi:10.1038/ncomms11470)
Supplement: Supplementary Information — Supplementary Figures 1-19, Supplementary Methods and Supplementary References [file ncomms11470-s1.pdf]

Supplementary Figures

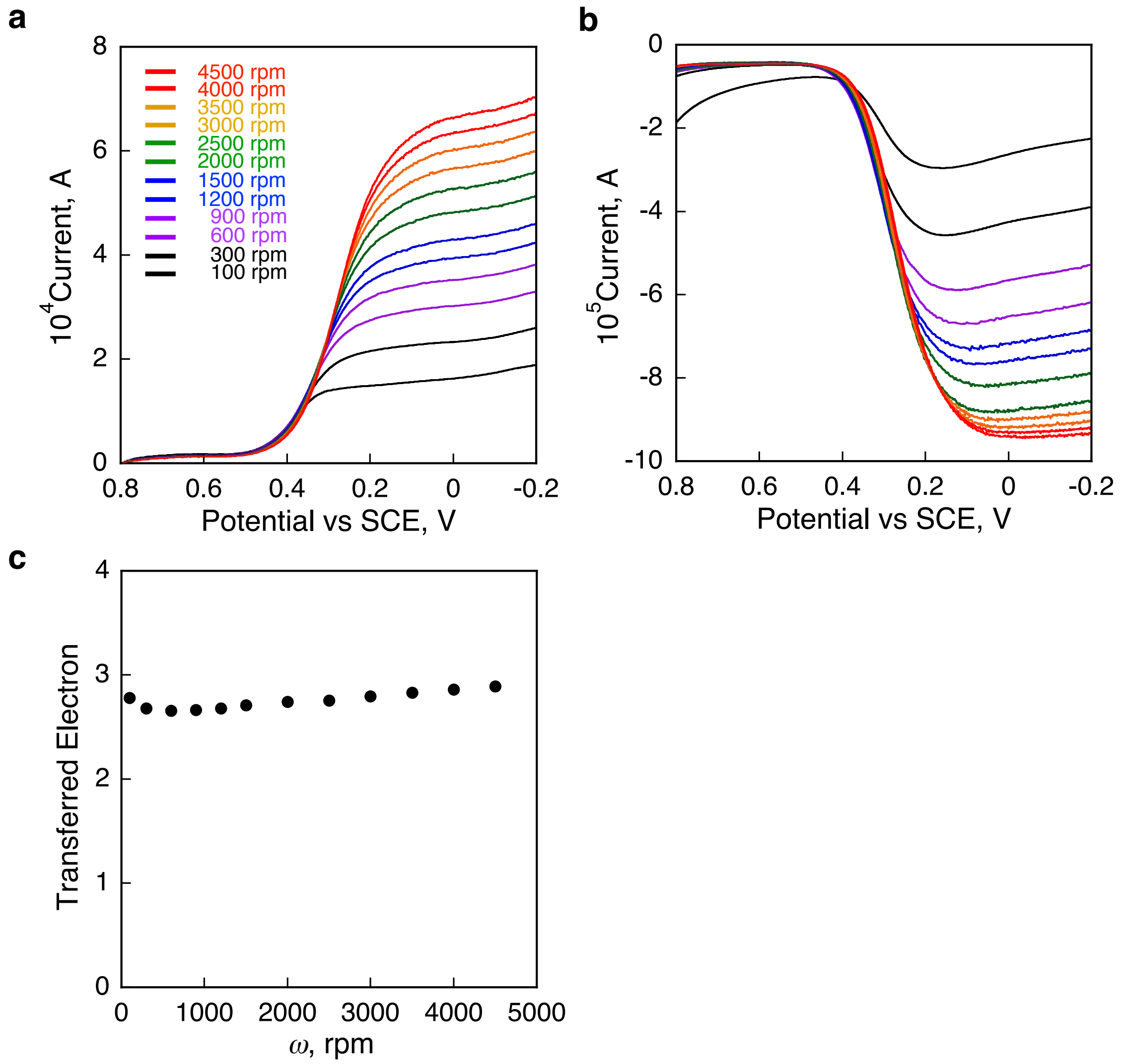

Supplementary Figure 1 | Determination of transferred electrons in the catalytic O_2 reduction with Co^{II}(Ch) deposited on electrode by RRDE Measurements. Linear sweep voltammograms (polarization curves) of an O_2-saturated aqueous $HClO_4$ solution (pH 1.3) recorded at a rotating-ring (Pt) and a disk (Co^{II}Ch/MWCNT/GC) electrode. (**a**) Currents at disk electrode for the reduction of O_2. (**b**) Currents at ring electrode for the reoxidation of H_2O_2 produced on the disk electrode with applied potential at 1.2 V vs SCE. (**c**) Plot of transferred electrons at 0 V vs rotation rate. Sweep rate: 5 mV s^{-1}.

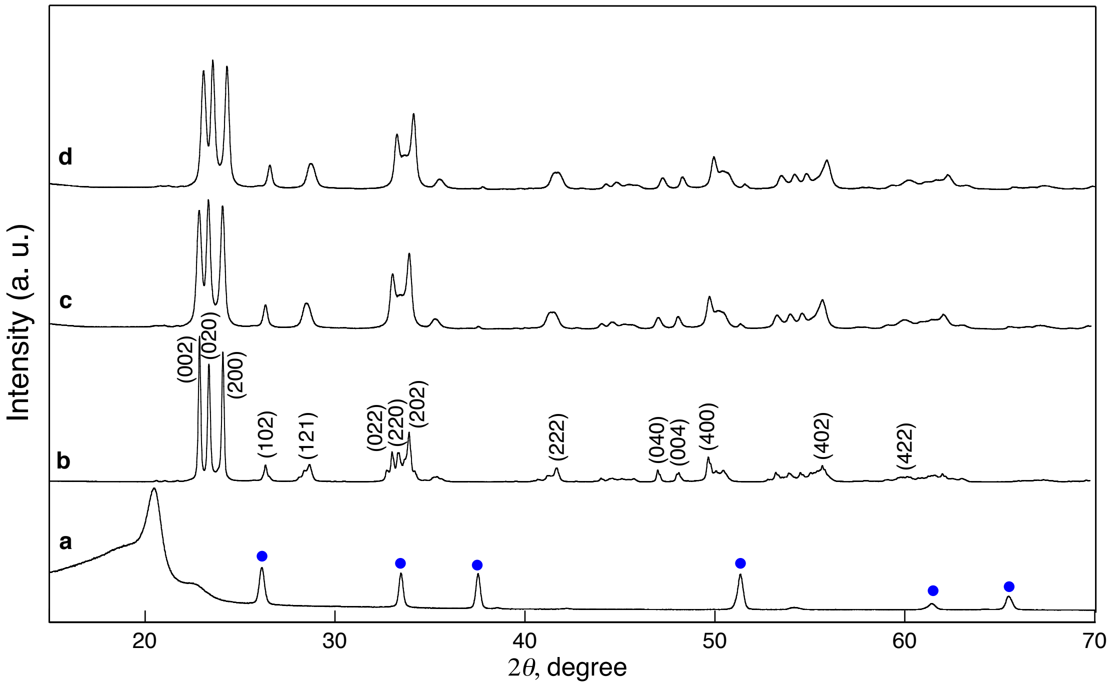

Supplementary Figure 2 | Powder X-ray diffraction pattern of the m-WO_3/FTO electrode (**a**) FTO electrode for the reference, (**b**) authentic sample of WO_3 for the reference, (**c**) as-prepared m-WO_3/FTO electrode before the photocatalytic reaction, (**d**) as-prepared m-WO_3/FTO electrode after the photocatalytic reaction for 24 h in pH1.3 seawater containing 0.1 M $NaClO_4$ under simulated 1 sun (AM 1.5G) illumination. Blue circles indicate the peaks derived from FTO electrode.

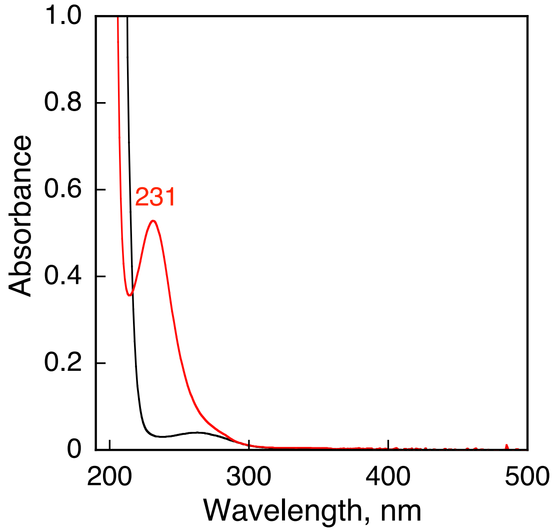

Supplementary Figure 3 | Absorption spectra of the reaction solution in the anode cell. The black and red lines show the spectra before and after the photocatalytic reaction for 24 h in pH 1.3 seawater containing 0.1 M $NaClO_4$ under simulated 1 sun (AM 1.5G) illumination.

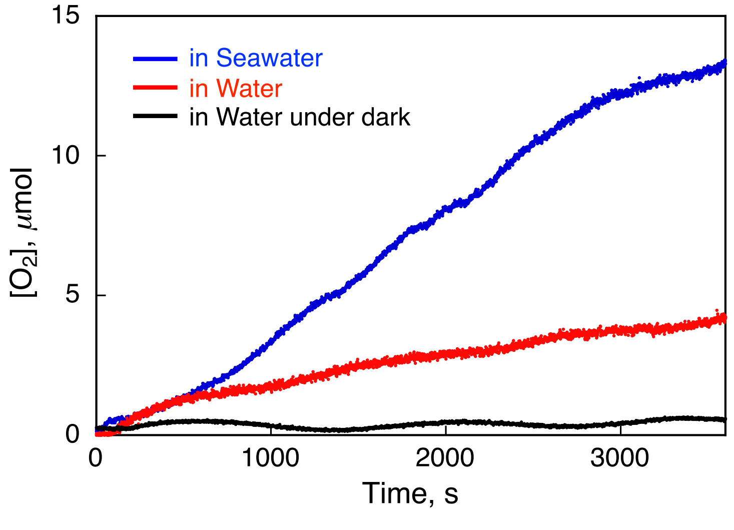

Supplementary Figure 4 | Photocatalytic evolution of O_2 in the two-compartment photoelectrochemical cell. Time courses of O_2 evolution with m-WO_3/FTO photoanode and Co^{II}(Ch)/CP cathode in an Ar-saturated pH 1.3 water (red) and pH 1.3 seawater (blue) under simulated 1 sun (AM 1.5G) illumination. Time course of O_2 concentration with m-WO_3/FTO photoanode and Co^{II}(Ch)/CP cathode in an Ar-saturated pH 1.3 water under dark is shown as black line.

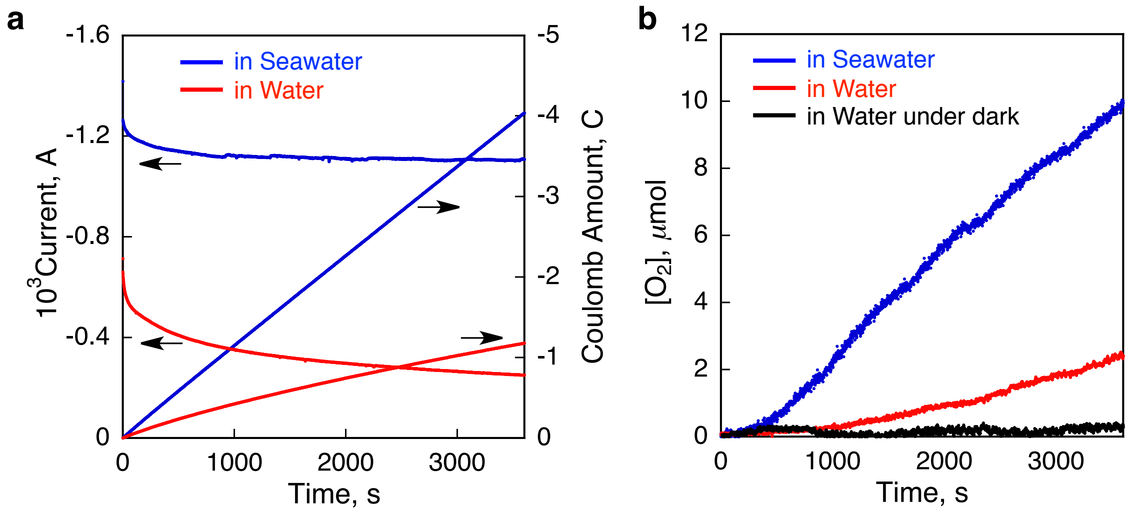

Supplementary Figure 5 | Photoelectrochemical performances of the m-WO_3/FTO electrode during water oxidation in water and in seawater. Time courses of (**a**) photocurrents, coulomb amount, and (**b**) O_2 evolution for the water oxidation with the m-WO_3/FTO electrode measured at 0.3 V (vs SCE) in an Ar-saturated pH 1.3 water (red) and pH 1.3 seawater (blue) containing 0.1 M $NaClO_4$ under simulated 1 sun (AM 1.5G) illumination. Time course of O_2 evolution with the m-WO_3/FTO electrode measured at 0.3 V (vs SCE) in an Ar-saturated pH 1.3 water under dark is shown as black line.

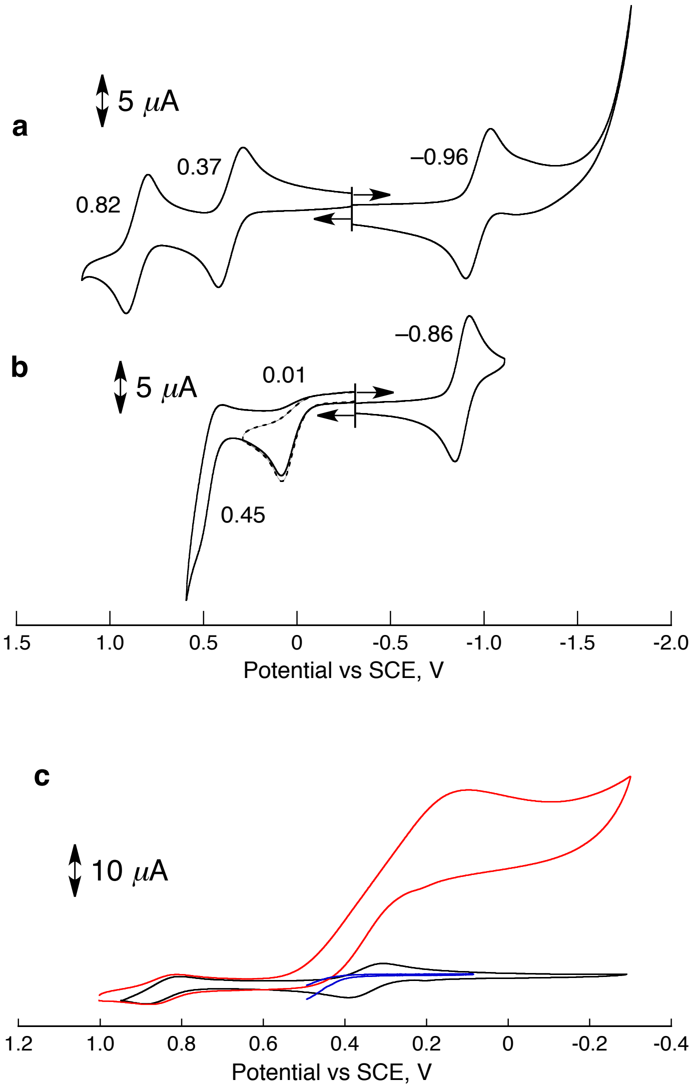

Supplementary Figure 6 | Effect of Cl^- on the redox property of Co^{II}(Ch) in PhCN. Cyclic voltammograms of deaerated PhCN solutions of Co^{II}(Ch) (1.0×10^{-3} M) recorded in the presence of $TBAPF_6$ (0.10 M) (**a**) without TBACl and (**b**) with TBACl (0.1 M). The sweep rate was 100 mV s^{-1}. (**c**) Cyclic voltammograms of Co^{II}(Ch) (1.0×10^{-3} M) in O_2-saturated PhCN containing $TBAPF_6$ (0.10 M) (red) and N_2-saturated PhCN containing $TBAPF_6$ (0.10 M) (black). The blue line shows the cyclic voltammogram of Co^{II}(Ch) (1.0×10^{-3} M) after the addition of TBACl (0.1 M) in O_2-saturated PhCN containing $TBAPF_6$ (0.10 M). The sweep rate was 100 mV s^{-1}.

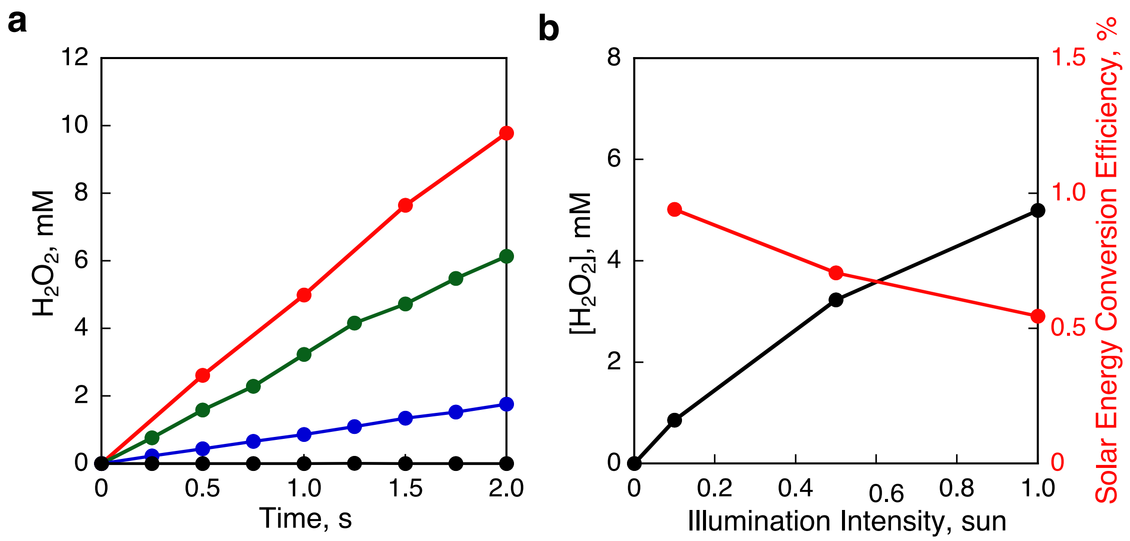

Supplementary Figure 7 | Dependence of the illumination intensity on the rate of the photocatalytic production of H_2O_2 and solar energy conversion efficiency. (**a**) Time courses of the photocatalytic production of H_2O_2 in the two-compartment cell composed of m-WO_3/FTO photoanode and Co^{II}(Ch)/CP cathode in pH 1.3 seawater containing 0.1 M $NaClO_4$ under different intensity of simulated illumination (AM 1.5G); 0 sun (black), 0.1 sun (blue) and 0.5 sun (green) and 1.0 sun (red). (**b**) Plots of the produced amount of H_2O_2 and solar energy conversion efficiency vs illumination intensity determined after the photocatalytic reaction for 1 h, respectively. The value of solar energy conversion efficiency was determined from the produced amount of H_2O_2 during initial 1h.

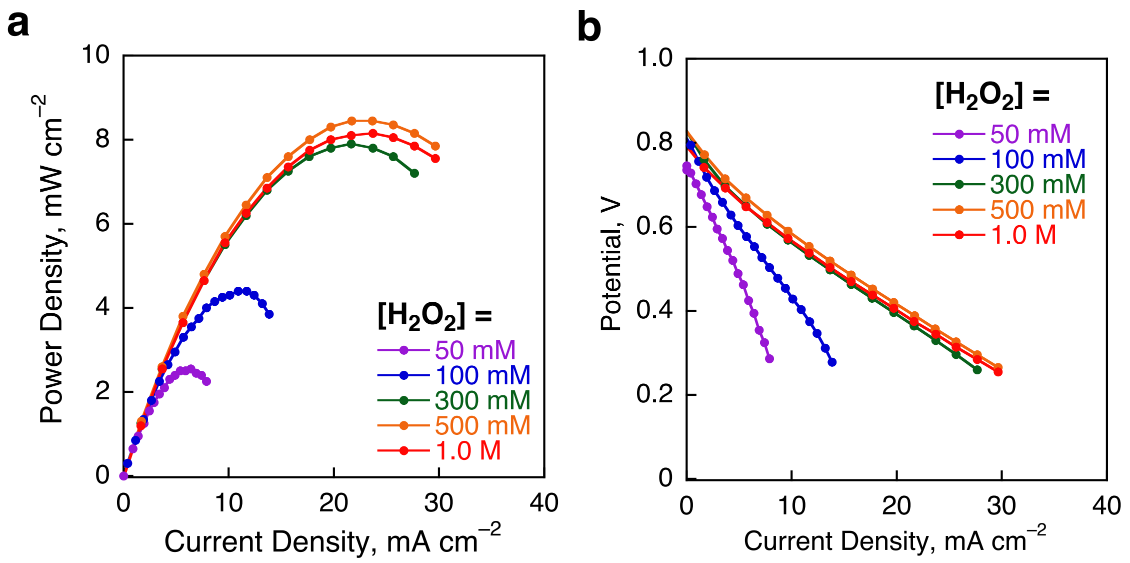

Supplementary Figure 8 | Generation of electrical energy in the one-compartment H_2O_2 fuel cell. (**a**) *I-P* and (**b**) *I-V* curves of the one-compartment H_2O_2 fuel cell with a Ni mesh anode and $Fe^{II}_3[Co^{III}(CN)_6]_2$/carbon cloth cathode in an aqueous $HClO_4$ (pH 1.0) solution containing various concentration of authentic H_2O_2 (50 mM ~ 1 M) and NaCl (1.0 M) as a supporting electrolyte.

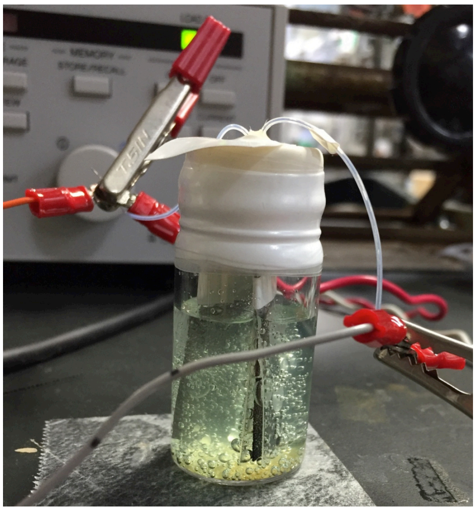

Supplementary Figure 9 | Digital picture of the H_2O_2 fuel cell. One-compartment H_2O_2 fuel cell with a Ni mesh anode and $Fe^{II}_3[Co^{III}(CN)_6]_2$/carbon cloth cathode in an aqueous H_2O_2 (0.3 M) solution containing $Sc(NO_3)_3{\bullet}4H_2O$ (0.1 M) and NaCl (1.0 M) as a supporting electrolyte.

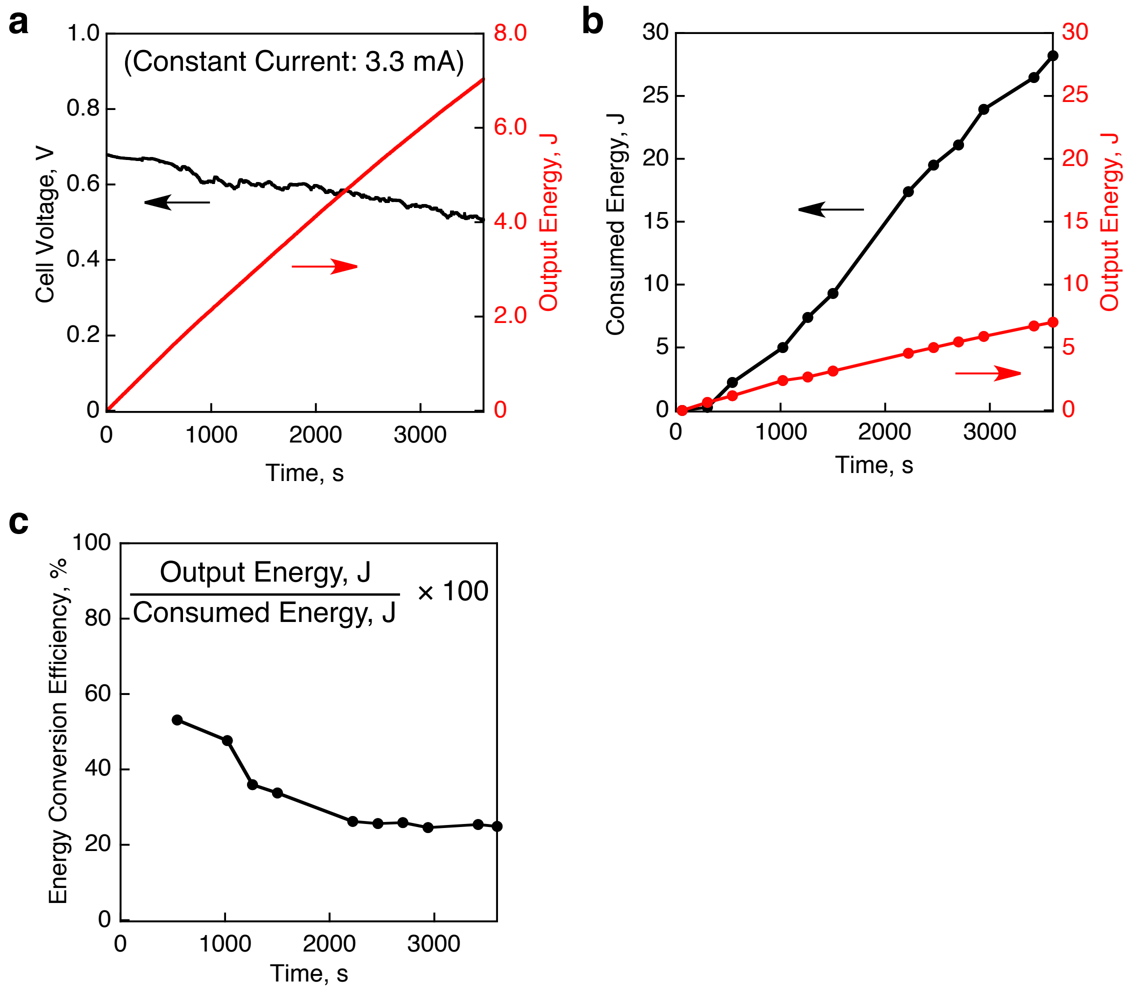

Supplementary Figure 10 | The energy conversion efficiency of H_2O_2 fuel cell. (**a**) Time courses of the cell voltage and the output energy of the one-compartment H_2O_2 fuel cell with a Ni mesh anode and $Fe^{II}_3[Co^{III}(CN)_6]_2$/carbon cloth cathode in an aqueous H_2O_2 (0.3 M) solution containing $Sc(NO_3)_3$•$4H_2O$ (0.1 M) and NaCl (1.0 M) as a supporting electrolyte. (**b**) Time courses of the consumed energy and output energy. (**c**) Time courses of the energy conversion efficiency of H_2O_2 fuel cell.

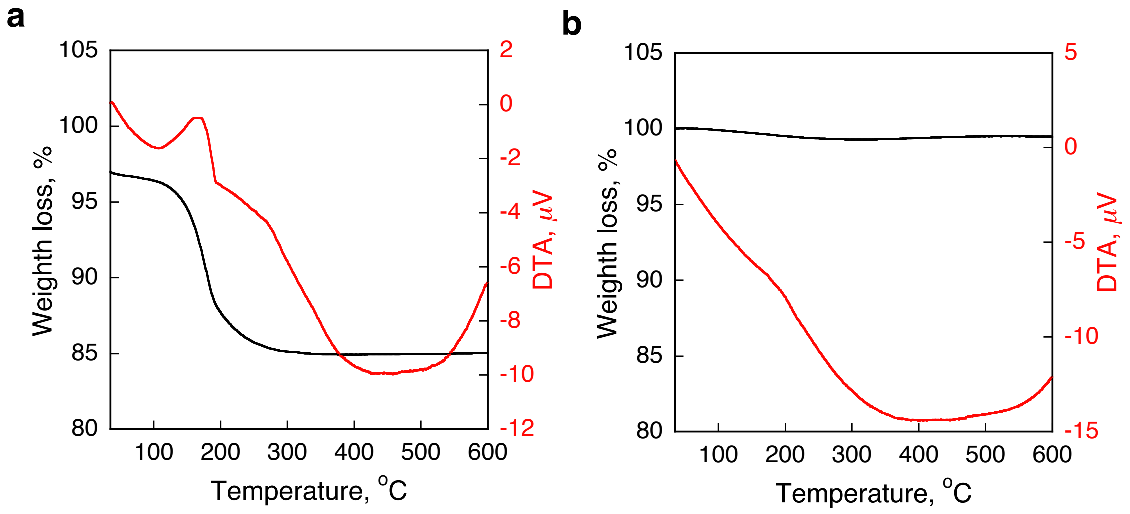

Supplementary Figure 11 | TG/DTA measurements. TG/DTA data of m-WO_3 dispersion consisting of 1 mL of water containing 50 mg of m-WO_3 and acetyl acetone (30 μL) and 1 drop of Triton X-100 (**a**) before and (**b**) after calcination at 400ºC with ramping rate of 2 ºC/min for 2 h under air. The black and red lines show TG curve and DTA curve, respectively.

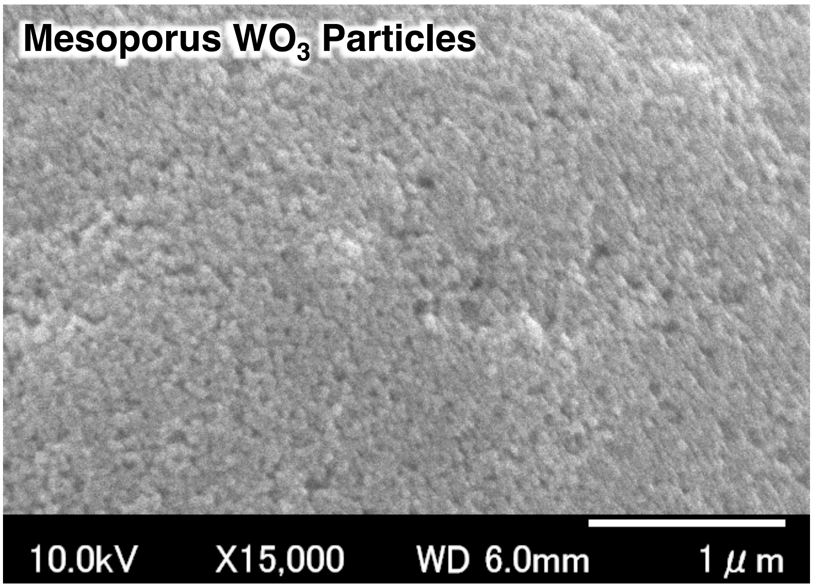

Supplementary Figure 12 | SEM image of m-WO_3/FTO electrode.

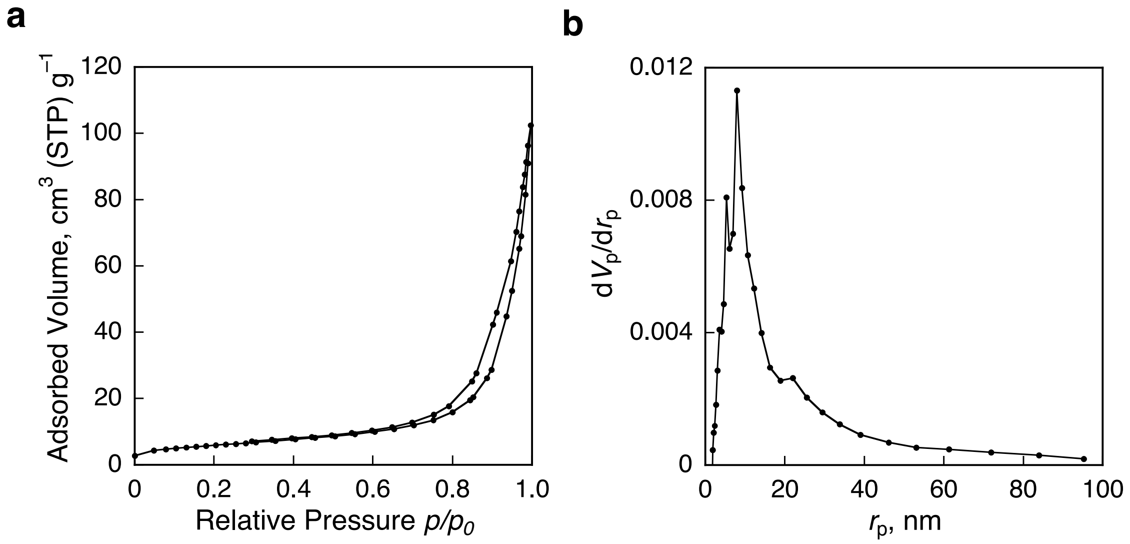

Supplementary Figure 13 | (a) Nitrogen adsorption-desorption isotherm and (b) BJH plot of m-WO_3.

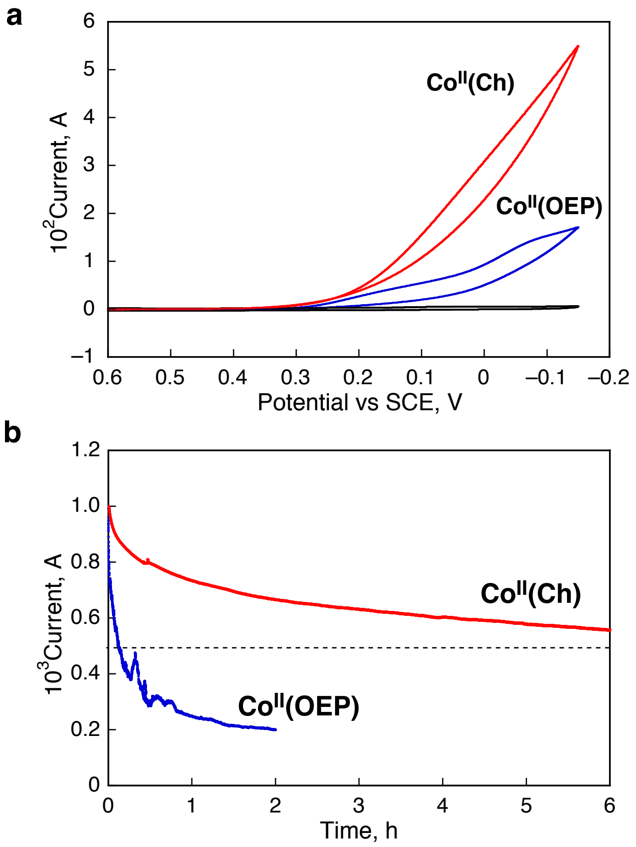

Supplementary Figure 14 | Electrochemical performances of Co^{II}(Ch)/CP and Co^{II}(OEP)/CP in the catalytic O_2 reduction. (**a**) Cyclic voltammograms of O_2-saturated aqueous $HClO_4$ (pH 1.3) solution containing 0.1 M $NaClO_4$ recorded at Co^{II}(Ch)/CP electrode (red) and Co^{II}(OEP)/CP electrode (blue). The black line shows cyclic voltammogram of N_2-saturated aqueous solution recorded at Co^{II}(Ch)/CP electrode.; Sweep rate: 20 mV s^{-1}. (**b**) Time courses of electrochemical reduction of O_2 with Co^{II}(Ch)/CP electrode (red) and Co^{II}(OEP)/CP electrode (blue) in O_2-saturated aqueous $HClO_4$ (pH 1.3) solution containing 0.1 M $NaClO_4$. The each applied potential (vs SCE) was adjusted to generate 1 mA of current at t = 0. The cyclic voltammogram of Co^{II}(Ch)/CP shows earlier onset potential at around 0.34 V (vs SCE) along with the rapid increase in catalytic current during negative-direction scan compared to that of Co^{II}(OEP)/CP (Supplementary Fig. 9a). The overpotential of two-electron reduction with Co^{II}(Ch)/CP was determined to be *ca.* 20 mV with respect to the theoretical redox potential for the two-electron reduction of O_2 in a pH 1.3 aqueous solution (E = 0.36 V vs SCE). The stability of these two electrodes was compared by applying bias potential adjusted to generate 1 mA as a starting current. The time course of Co^{II}(Ch)/CP shows higher electrocatalytic current with superior stability compared to the case of Co^{II}(OEP)/CP, where the half-life period of current with Co^{II}(Ch)/CP is more than 36 times longer than that of Co^{II}(OEP)/CP (Supplementary Fig. 9b).

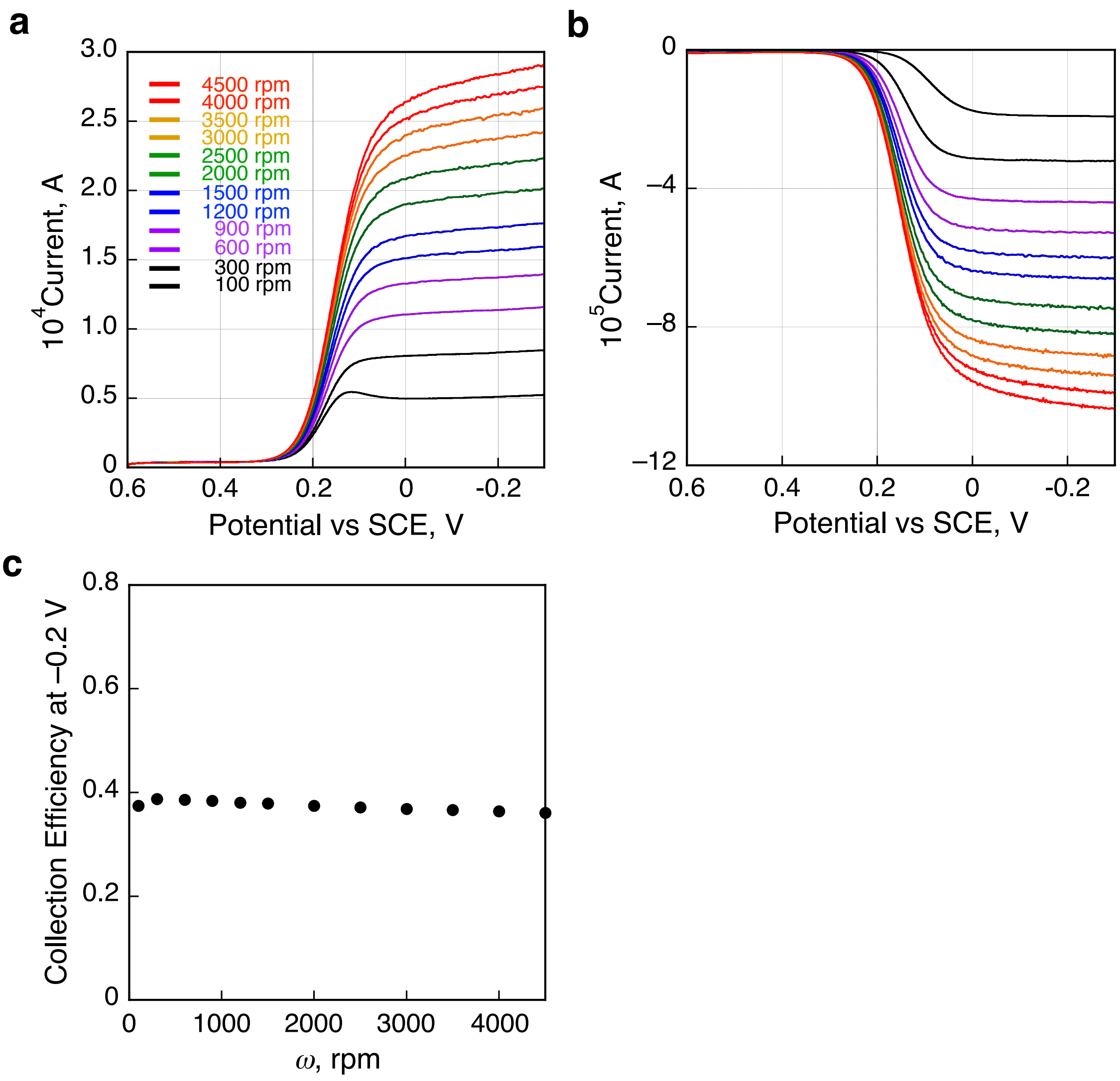

Supplementary Figure 15 | Determiniation of correction efficincy of RRDE with $K_3[Fe^{III}(CN)_6]$. Linear sweep voltammograms (polarization curves) of a N_2-saturated aqueous solution of $K_3[Fe^{III}(CN)_6]$ (2.0×10^{-3} M) in the presence of KNO_3 (0.5 M) recorded at a rotating-ring (Pt) and disk (GC) with physisorbed MWCNT electrode. (**a**) Currents at disk electrode for the reduction of $K_3[Fe^{III}(CN)_6]$. (**b**) Currents at ring electrode for the reoxidation of reduced $K_2[Fe^{II}(CN)_6]$ species produced on the disk electrode with applied potential at 0.6 V vs SCE. Sweep rate: 5 mV s^{-1}. (**c**) Plots of collection efficiency at –0.2 V vs rotation rate.

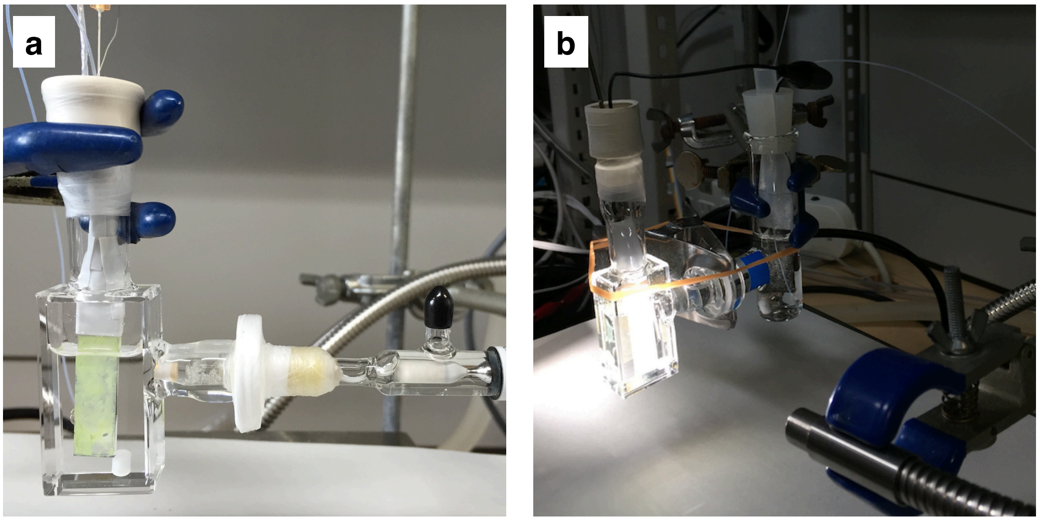

Supplementary Figure 16 | Digital picture of the as-fabricated device. (**a**) A photoelectochemical cell composed of the as prepared m-WO_3/FTO electrode, a platinum coil counter electrode, and a SCE reference electrode. (**b**) A photoelectochemical cell connected with a pyrex cathode cell through a Nafion membrane.

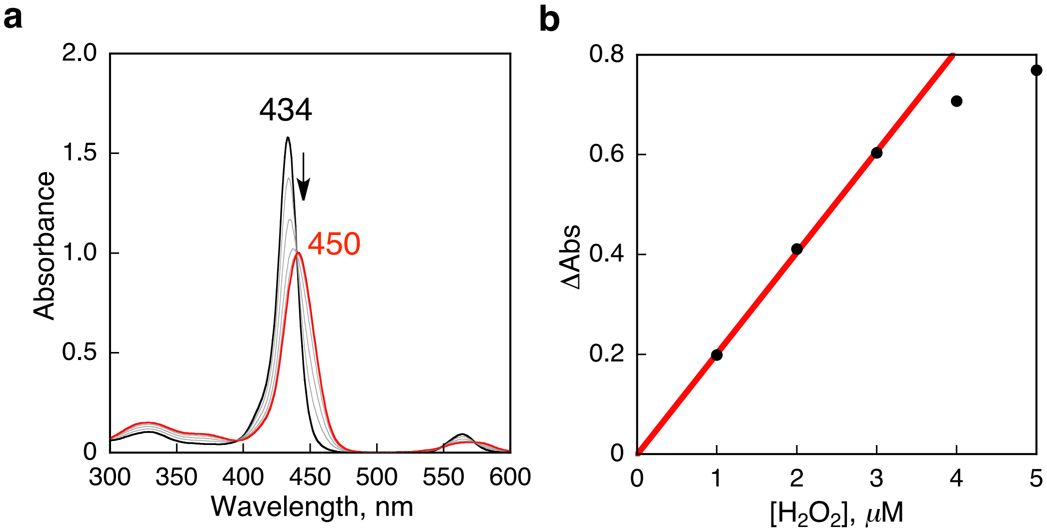

Supplementary Figure 17 | Calibration curve of Ti-TPyP reagent for the detection of H_2O_2. (**a**) Absorption spectral changes of the Ti-TPyP reagent in the reaction of Ti-TPyP (5.0×10^{-6} M) upon addition of H_2O_2 [0 μM (black line) and 5 μM (red line)] in an aqueous solution containing $HClO_4$ (0.5 M). (**b**) Plot of the absorbance change at λ = 434 nm versus the concentration of H_2O_2.

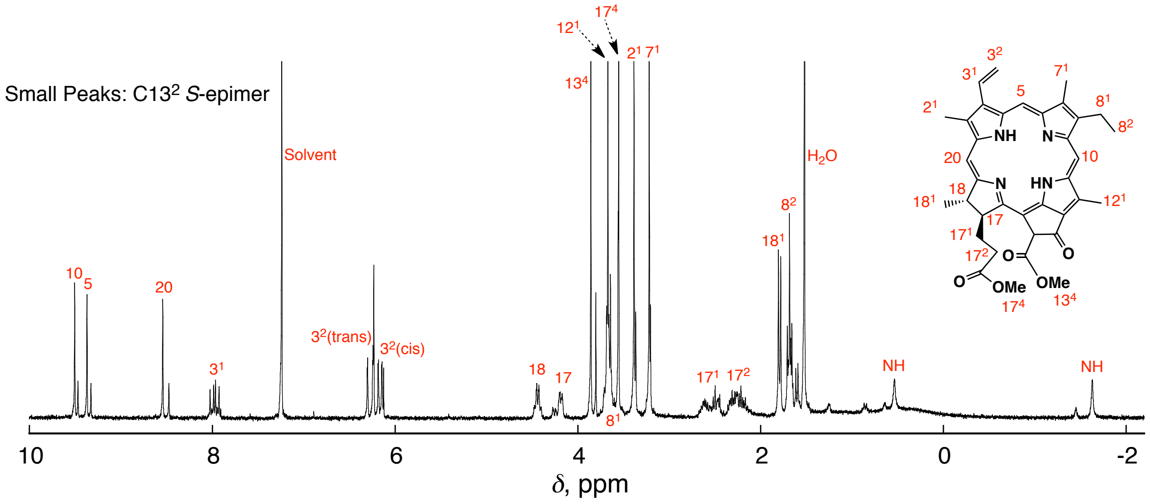

Supplementary Figure 18 | ^{1}H NMR spectrum of pheophytin *a* in $CDCl_3$ (300 MHz, 298 K).

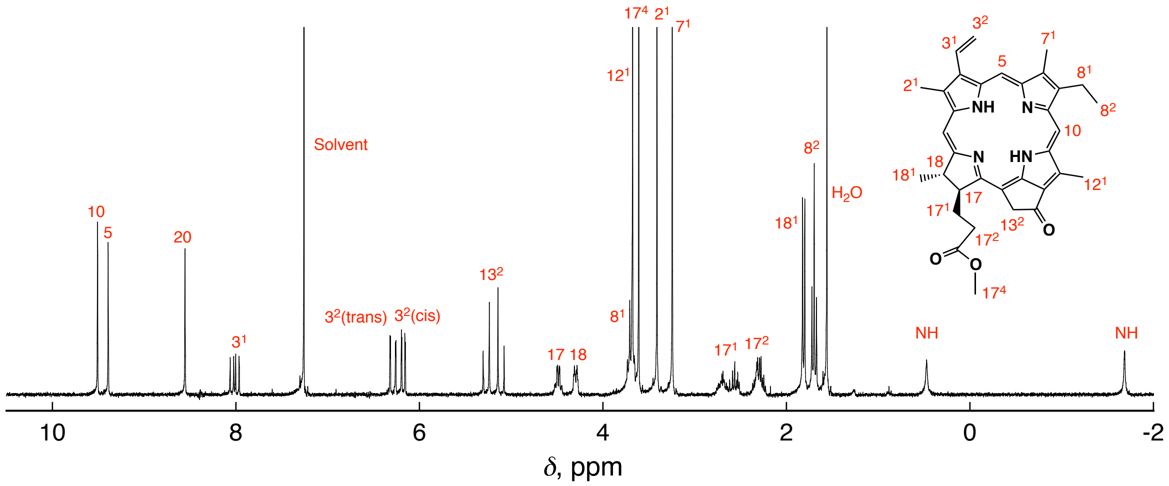

Supplementary Figure 19 | ^{1}H NMR spectrum of H_2(Ch) in $CDCl_3$ (300 MHz, 298 K).

Supplementary Methods

Synthesis of Free Base Chlorin (H_2(Ch)). H_2(Ch) was synthesised by two-step reaction: extraction of pheophytin *a* from *Spirulina* algae and subsequent decarboxylation of the methoxycarbonyl group (position C-13^2) according to published procedures.[S1-5]. *Spirulina* algae was purchased from Japan Alge Corp. MeOH (500 mL) was poured to *Spirulina* powder (300 g) several times to extract organic compounds. After removal of insoluble residues by filtration, acetic acid (15 mL) was added to the filtrate to remove magnesium ion. The resulting solution was evaporated to reduce the volume to 100 mL and neutralised with an aqueous solution of saturated $NaHCO_3$. pheophytin *a* was extracted by adding CH_2Cl_2 to the mixture and the CH_2Cl_2 phase was washed with distilled water several times. After the solvent was evaporated, the concentrated solution was purified by silica gel column chromatography. The first yellow and red bands corresponding to carotenoids were eluted with CH_2Cl_2, and then a black band corresponding to the product (pheophytin *a*) was eluted with 20% ethyl acetate in CH_2Cl_2. The product was recrystallized from CH_2Cl_2/hexane to give pheophytin *a* as a dark blue solid (2.5g). Both 13^2 *R*-epimer and a small amount of 13^2 *S*-epimer were formed. ^{1}H NMR ($CDCl_3$, 300 MHz, pheophytin *a*) = 5.0×10^{-3} M): δ –1.63 (1H, br, s, -NH), 0.53 (1H, br, s, -NH), 1.53 (s, H_2O), 1.68 (3H, t, J = 7.5 Hz, 8^2-CH_3), 1.79 (3H, d, J = 8.1 Hz, 18^1-CH_3), 2.17-2.32 (2H, m, 17^2-CH_2-), 2.55-2.65 (2H, m, 17^1-CH_2-), 3.22 (3H, s, 7^1-CH_3), 3.38 (3H, s, 2^1-CH_3), 3.55 (3H, s, 17^4-CO_2CH_3), 3.69 (3H, s, 12^1-CH_3), 3.70 (2H, q, J = 7.8 Hz, 8^1-CH_2-), 3.85 (3H, s, 13^4-CO_2CH_3), 4.16-4.20 (1H, m, 17-H), 4.42-4.45 (1H, m, 18-H), 6.17 (1H, dd, J = 10.2, 1.5 Hz, 3^2=CH_2 *cis*), 6.24 (1H, s, 13^2-CH-), 6.26 (1H, dd, J = 17.2, 1.5 Hz, 3^2=CH_2 trans), 7.24 (s, solvent), 7.97 (1H, dd, J = 18.4, 11.7 Hz, 3^1-CH=), 8.54 (1H, s, 20-H), 9.37 (1H, s, 5-H), 9.51 (1H, s, 10-H) (Supplementary Fig. 18).

The obtained pheophytin *a* (1.0 g) was dissolved in 2,4,6-collidine, and the reaction mixture was refluxed for 3 h under dark and nitrogen. After the solvent was evaporated at reduced pressure, the resulting solution was dissolved in 10% sulphuric acid in CH_3OH, and the reaction mixture was stirred for 12 h under dark and nitrogen at room temperature. The resulting solution was neutralised with the aqueous solution of saturated $NaHCO_3$. H_2(Ch) was extracted by adding CH_2Cl_2 to the mixture and the CH_2Cl_2 phase was washed with distilled water several times. After the solvent was evaporated, the crude solid was purified by silica gel column chromatography. A black

band corresponding to the product ($H_2(Ch)$) was eluted with the mixed solution (CH_3OH/hexane/$CHCl_3$ [1/8/20 (v/v/v)]). The product was recrystallized from CH_2Cl_2/hexane to give $H_2(Ch)$ as a dark blue solid (660 mg). ^1H NMR ($CDCl_3$, 300 MHz, $H_2(Ch) = 5.0 \times 10^{-3}$ M): δ –1.68 (1H, br, s, -NH), 0.47 (1H, br, s, -NH), 1.51 (s, H_2O), 1.70 (3H, t, J = 7.7 Hz, 8^2-CH_3), 1.80 (3H, d, J = 6.9 Hz, 18-CH_3), 2.24-2.35 (2H, m, 17^2-CH_2-), 2.51-2.70 (2H, m, 17^1-CH_2-), 3.25 (3H, s, 7^1-CH_3), 3.41 (3H, s, 2^1-CH_3), 3.61 (3H, s, 17^4-CO_2CH_3), 3.68 (3H, s, 12^1-CH_3), 3.70 (2H, q, J = 7.7 Hz, 8^1-CH_2-), 4.30 (1H, m, 17-H), 4.49 (1H, m, 18-H), 5.18 (2H, q, J = 20.1 Hz, 13^2-CH_2-), 6.17 (1H, dd, J = 10.2, 1.5 Hz, 3^2=CH_2 *cis*), 6.29 (1H, dd, J = 17.5, 1.5 Hz, 3^2=CH_2 trans), 7.26 (s, solvent), 8.01 (1H, dd, J = 17.5, 11.3 Hz, 3^1-CH=), 8.56 (1H, s, 20-H), 9.40 (1H, s, 5-H), 9.51 (1H, s, 10-H) (Supplementary Fig. 19).

Synthesis of Cobalt Chlorin (Co(Ch)) Cobalt chlorin was synthesised by following the published method.[6] A mixture of Free base chlorin $H_2(Ch)$ (100 mg), $Co(CH_3CO_2)_2$•$4H_2O$ (200 mg) and CH_3COONa (500 mg) in 150 mL of $CHCl_3$/CH_3OH [2/1 (v/v)] was refluxed under dark and nitrogen for 6 h. The reaction mixture was poured into distilled water, extracted with CH_2Cl_2, washed several times with distilled water and dried over Na_2SO_4. The product was purified by silica gel column chromatography using a solution (acetone/$CHCl_3$ [1/10 (v/v)]) as an eluent and recrystallized from CH_2Cl_2/hexane to give $Co^{II}(Ch)$ as a green solid.

Synthesis of mesoporus WO_3 (m-WO_3). m-WO_3 was synthesised according to the published method.[7] Pluronic P-123 (1 g) and WCl_6 (3.96 g, 0.01 mol) were dissolved in 10 mL of EtOH with vigorous stirring in ice bath. The resulting dark blue solution was gelled in an open Petri dish for aging at 40ºC in air for 3days. The resulting dark blue solid was calcinated at 400ºC with ramping rate of 0.5 ºC/min for 5h under air to remove surfactant species. The obtained yellow solid was ground with mortar.

Synthesis of $Fe^{II}_3[Co^{III}(CN)_6]_2$. $Fe^{II}_3[Co^{III}(CN)_6]_2$ was synthesised by following the published method.[8] An aqueous solution of $FeSO_4$•$7H_2O$ (0.18 M, 10 mL) was slowly added to an aqueous solution of $K_3[Co^{III}(CN)_6]$ (0.12 M, 10 mL) with vigorous stirring. The formed precipitate was collected by filtration and washed with pure water several times. The resulting precipitate was dried at 60ºC for 12 hours.

Supplementary References

1. Wasielewski, M. R. & Svec, W. A. Synthesis of covalently linked dimeric derivatives of chlorophyll-*a*, pyrochlorophyll-*a*, chlorophyll-*b*, and bacteriochlorophyll-*a*. *J. Org. Chem.* **45**, 1969-1974 (1980).
2. Zheng, G. *et al.* Low-density lipoprotein reconstituted by pyropheophorbide cholesteryl oleate as target-specific photosensitizer. *Bioconjugate Chem.* **13**, 392-396 (2002).
3. Arian, D., Clo, E., Gothelf, K. V. & Mokhir, A. A nucleic acid dependent chemical photocatalysis in live human cells. *Chem. Eur. J.* **16**, 288-295 (2010).
4. Ishigure, S. *et al.* Peroxide decoloration of CI Acid Orange 7 catalyzed by manganese chlorophyll derivatives at the surfaces of micelles and lipid bilayers. *Langmuir* **26**, 7774-7782 (2010).
5. Paolesse, R. *et al.* Stepwise syntheses of bisporphyrins, bischlorins, and biscorroles, and of porphyrin-chlorin and porphyrin-corrole heterodimers. *J. Am. Chem. Soc.* **118**, 3869-3882 (1996).
6. Mase, K., Ohkubo, K. & Fukuzumi, S. Efficient two-electron reduction of dioxygen to hydrogen peroxide with one-electron reductants with a small overpotential catalyzed by a cobalt chlorin complex. *J. Am. Chem. Soc.* **135**, 2800-2808 (2013).
7. Yang, P., Zhao, D., Margolese, D. I., Chmelka, B. F. & Stucky, G. D. Generalized syntheses of large-pore mesoporous metal oxides with semicrystalline frameworks. *Nature* **396**, 152-155 (1998).
8. Yamada, Y., Yoneda, M. & Fukuzumi, S. A robust one-compartment fuel cell with a polynuclear cyanide complex as a cathode for utilizing H_2O_2 as a sustainable fuel at ambient conditions. *Chem. Eur. J.* **19**, 11733-11741 (2013).
